# Prognostic value of systemic inflammatory response index for acute kidney injury and the prognosis of pediatric patients in critical care units

**DOI:** 10.1371/journal.pone.0306884

**Published:** 2024-08-29

**Authors:** Danchi Lu, Lijuan Tu, Yugang Hu, Xiaofang Cai

**Affiliations:** 1 Department of Emergency, Wuhan Children’s Hospital, Tongji Medical College, Huazhong University of Science & Technology, Wuhan, China; 2 Department of Ultrasound Imaging, Renmin Hospital of Wuhan University, Wuhan, China; Metrohealth Medical Center, UNITED STATES OF AMERICA

## Abstract

**Background:**

We proposed a link between the first systemic inflammatory response index (SIRI) and acute kidney injury (AKI), as well as the prognosis of pediatric patients in intensive care units (PICU).

**Methods:**

This study comprised 5114 children from the pediatric-specific intensive care (PIC) database. SIRI was estimated as a neutrophil monocyte lymphocyte ratio. All patients were arbitrarily allocated to the training set (n = 3593) and the validation cohort (n = 1521) and divided into two groups depending on their SIRI levels. The diagnostic value of SIRI for pediatric ICU patients was subsequently determined using LASSO regression models.

**Results:**

After controlling for additional confounding variables in the training set, the higher SIRI value (≥ 0.59) had a greater risk of AKI (adjusted odds ratio, OR, 3.95, 95% confidence interval, 95%CI, 2.91–5.36, P<0.001) and in-hospital mortality (hazard ratio, HR, 5.01, 95%CI 2.09–12.03, P<0.001). Similar findings were discovered in the validation set. Furthermore, the suggested nomogram derived from SIRI and other clinical metrics showed outstanding calibration capability as well as therapeutic usefulness in both groups.

**Conclusions:**

SIRI is a reliable and useful factor for AKI and fatality in pediatric ICU patients, and the proposed nomogram based on SIRI yields an appropriate prediction value for critically sick pediatric patients.

## Introduction

Despite advances in the mechanism and medical technology for diseases in intensive care units (ICU), acute kidney injury (AKI) remains one of the most prevalent adverse events for both adult and pediatric populations, resulting in greater deaths and morbidity, more time in the hospital, and more expensive health care [1–3]. Unlike in older individuals, AKI in pediatric ICU (PICU) patients tends to be defined by a complicated disease progression, and unusual signs & symptoms that may worsen at any time, making it potentially life-threatening [4]. As a result, doctors have to focus on noticing patients at high risk of AKI using biological indicators or algorithms for prediction to handle these patients successfully and offer swift strategies, which will enhance their prognosis [5–7].

The systemic inflammatory response index (SIRI), defined as neutrophil monocyte/lymphocyte count, has proven to be incredibly useful as a diagnostic and prognostic tool for patients with many disorders, including renal ailments [8–10]. Several studies have also shown that SIRI may be a potential indicator for AKI in adult patients with various illnesses [11–13]. Furthermore, because children may have distinct developmental concerns and varied reactions to treatments and AKI recovery, the efficiency of SIRI and AKI must be thoroughly investigated, particularly in pediatric patients. The relationship between the SIRI and the assessment and therapeutic parameters of AKI in children, on the other hand, had never been studied before. As a result, the goal of this study was to investigate the predictive value of SIRI for AKI and the prognosis of patients in the PICU; Additionally, a predictive nomogram for AKI was generated to offer an affordable biomarker identification for medical professionals to promptly recognize AKI and optimize the clinical outcomes.

## Materials and methods

### Data source

The basis of this study was the Pediatric-Specific Intensive Care Database (PIC) [14], an anonymous and broad medical record featuring routine medical documents of over 13941 ICU admissions from the Children’s Hospital, Zhejiang University School of Medicine, from 2010 to 2018. This hospital’s Institutional Review Committee accepted the project and exempted it from getting patient informed consent.

### Selection of participants

**Fig 1:**
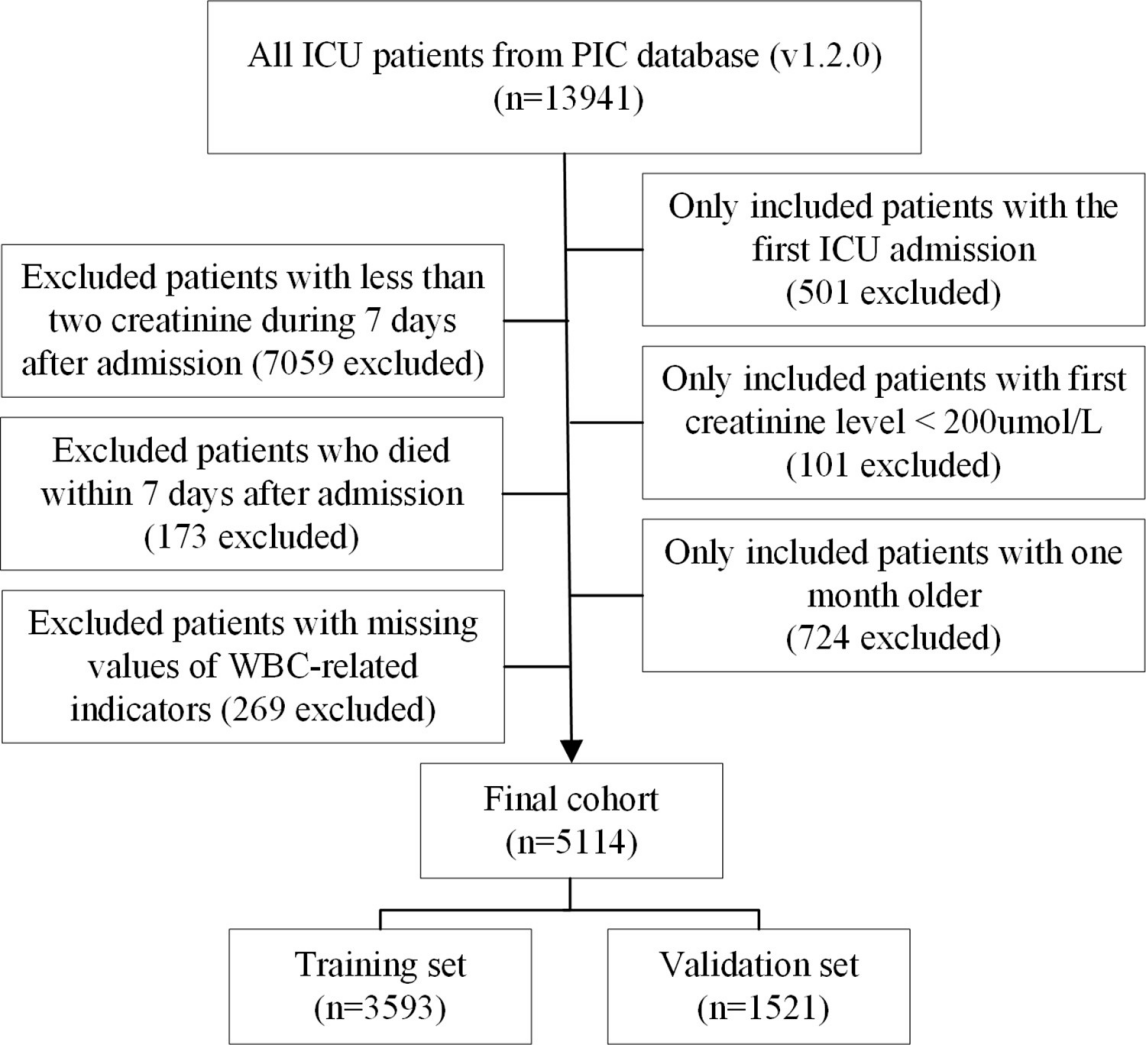
The flow chart of this study.

This research included all participants in the PIC database. Elimination criteria comprised children who had multiple ICU hospitalizations, died within seven days of admission, and had <1 creatinine measurement within seven days of ICU admission. We additionally eliminated individuals who were younger than one month old and had a first creatinine level of more than 200 mol/L due to the pROCK criterion. Furthermore, we eliminated individuals with missing neutrophil, monocyte, or lymphocyte counts. With this criteria, 5114 pediatric patients were included in the study, with the training set (n = 3593) and the validation cohort (n = 1521) being allocated at randomness (Fig 1).

### Data collection

PostgreSQL software (version 9.6, https://www.postgresql.org/) was employed to gather the information that was extracted, which comprised patients’ information such as age, gender, type of ICU admission, initial laboratory findings following ICU admission, and complications (including anemia and sepsis).

SIRI was estimated as a neutrophil monocyte lymphocyte ratio. Anemia was defined as having an initial hemoglobin level of less than 90g/L at 1–4 months old, 100g/L at 4–6 months old, 110g/L at 6–60 months old, 115g/L at 60–144 months old, and 120g/L at older than 144 months old [15]. We defined sepsis using the International Classification of Diseases (ICD-10) 10 codes.

### Outcomes

According to the pROCK specifications [16], the primary outcomes were the occurrence and seriousness of AKI, which implies that serum creatinine (SCr) increased by 0.2mg/dL or 1.3 times within 7 days. Moreover, the stages of AKI were as follows: Stage 1, SCr rise ≥ 0.2–0.4 mg/dL and ≥ 1.3–1.59 times the baseline SCr; stage 2, SCr rise ≥ 0.5–0.9 mg/dL and ≥ 1.6–2.19 times the baseline SCr; stage 3, SCr rise ≥ 1.0 mg/dL and ≥ 2.2 times the baseline SCr and the additional goals of this study included the use of vasopressors, sepsis, length of ICU stay, length of hospital stay, and in-hospital mortality.

### Statistical analysis

R (version 4.1.0) was used for all of the analysis. The receiver operator characteristic curve (ROC) was used to ensure the best cutoff value for all AKI parameters. In light of our expectation that the interaction between SIRI and the probability was non-linear, we investigated logistic models alongside restricted cubic splines (RCS), with four knots (5th, 35th, 65th, and 95th percentiles) for SIRI, adjusting for all of the covariates listed above, to more adaptable model the association of baseline SIRI values with AKI. Furthermore, we utilized a likelihood ratio test to investigate nonlinearity by contrasting the simulation model with only the linear portion and the cubic spline terms. For the identification of important predictors for AKI in PICU patients, the least absolute shrinkage and selection operator (LASSO) modeling was applied. Then, logistic regression analysis with multiple variables was performed to create a nomogram for predicting AKI in pediatric patients. We examined the effects of SIRI on AKI or clinical outcomes accounting for potential confounders by using three different models: (1) model I, adjusting for age, gender, and ICU category; (2) adjusting for model 1 plus for model I plus comorbidities (anemia and sepsis); 3) adjusting for model 2 plus laboratory results. The predictive efficacy of the AKI predictive nomogram was evaluated using calibration plots and decision curve analysis (DCA). A P < 0.05 was considered significant.

## Results

### Characteristics of all patients

This research ultimately included 5114 patients, comprising 2788 male (54.5%) and 2326 females (45.5%), with an average age of 28.3 ± 11.3 (1–214) months. All patients were allocated amongst two distinct groups unplanned: the training cohort (n = 3593) and the validation cohort (n = 1521). Table 1 contains the fundamental characteristics.

**Table 1:** The baseline characteristics of all patients.

Characteristics	Training set (n = 3593)			Internal validation set (n = 1521)		
	No AKI (n = 2633)	AKI (n = 960)	P value	No AKI (n = 1099)	AKI (n = 422)	P value
Age, months	37.5 ± 10.1	41.6 ± 11.4	0.014	35.1 ± 11.2	44.0 ± 11.9	<0.001
<12	1036 (39.3)	322 (33.5)		443 (40.3)	152 (36.0)	
12–60	989 (37.6)	392 (40.8)		421 (38.3)	148 (35.1)	
60–120	399 (15.2)	151 (15.7)		164 (14.9)	77 (18.2)	
>120	209 (7.9)	95 (9.9)		71 (6.5)	45 (10.7)	
Gender, male, n (%)	1439 (54.7)	526 (54.8)	0.971	598 (54.4)	225 (53.3)	0.744
ICU category, n (%)			<0.001			<0.001
CICU	1050 (39.9)	365 (38.0)		460 (41.9)	167 (39.6)	
PICU	469 (17.8)	313 (32.6)		168 (15.3)	139 (32.9)	
SICU	551 (20.9)	153 (15.9)		240(21.8)	64 (15.2)	
GICU	563 (21.4)	129 (13.4)		231 (21.0)	52 (12.3)	
Comorbidities, n (%)						
Anemia	795 (30.2)	307 (32.0)	0.324	300 (27.3)	141 (33.4)	0.022
Sepsis	611 (23.2)	171 (17.8)	0.002	207 (18.8)	100 (23.7)	<0.001
Usage of vasopressors, n (%)	404 (15.3)	122 (12.7)	0.117	163 (14.8)	49 (11.6)	0.123
Laboratory values						
White blood cell, × 10^9^/L	10.2 ± 4.3	11.4 ± 4.9	0.005	10.1 ± 4.7	14.5 ± 6.2	0.001
Hemoglobin, g/L	112.8 ± 20.9	115.2 ± 23.1	0.003	113.9 ± 21.1	114.9 ± 22.9	0.389
Platelet, × 10^9^/L	320.2 ± 109.7	295.4 ± 90.5	<0.001	317.6 ± 112.9	309.2 ± 114.4	0.304
SIRI	1.3 ± 0.5	2.6 ± 1.1	<0.001	1.2 ± 0.4	2.6 ± 0.7	<0.001
Albumin, g/L	40.9 ± 6.3	40.4 ± 6.9	0.095	41.2 ± 6.2	40.3 ± 6.9	0.012
Total bilirubin, μmol/L	17.0 ± 7.3	21.2 ± 10.5	0.008	17.2 ± 9.1	22.0 ± 10.7	0.046
Anion gap, mmol/L	7.9 ± 2.9	8.8 ± 3.5	<0.001	8.0 ± 3.1	8.9 ± 3.3	0.009
Bicarbonate, mEq/L	22.6 ± 3.2	22.1 ± 3.6	<0.001	22.7 ± 3.5	22.1 ± 3.8	0.006
BUN, mmol/L	4.0 ± 1.4	4.4 ± 1.5	<0.001	4.1 ± 1.6	4.5 ± 1.0	<0.001
Glucose, mmol/L	6.6 ± 2.3	7.0 ± 3.1	0.001	6.5 ± 2.1	6.9 ± 2.7	0.014
Calcium, mmol/L	1.7 ± 0.7	1.6 ± 0.6	<0.001	1.7 ± 0.7	1.6 ± 0.6	<0.001
Potassium, mmol/L	3.8 ± 0.7	3.8 ± 0.7	0.693	3.8 ± 0.6	3.8 ± 0.7	0.892
Sodium, mmol/L	137.4 ± 5.3	137.9 ± 5.2	0.012	136.8 ± 4.9	135.7 ± 5.0	0.672
CRP, mg/L	20.9 ± 10.6	28.4 ± 10.6	0.027	23.5 ± 10.7	28.0 ± 12.5	0.019
Lactate acid, mmol/L	1.9 ± 0.7	2.4 ± 0.9	<0.001	2.0 ± 0.8	2.2 ± 0.9	0.026
AKI stages, n (%)						
Stage I	-	804 (83.8)	-	-	346 (82.0)	-
Stage II	-	85 (8.9)	-	-	35 (8.3)	-
Stage III	-	71 (7.3)	-	-	41 (9.7)	-

AKI, acute kidney injury, CICU, cardiac intensive care unit, PICU, pediatric intensive care unit; SICU, surgery intensive care unit, GICU, general intensive care unit, SIRI, systemic inflammatory response index, BUN, blood urea nitrogen, CRP, C-reactive protein.

### The association between SIRI and AKI in PICU patients in the training set

**Fig 2:**
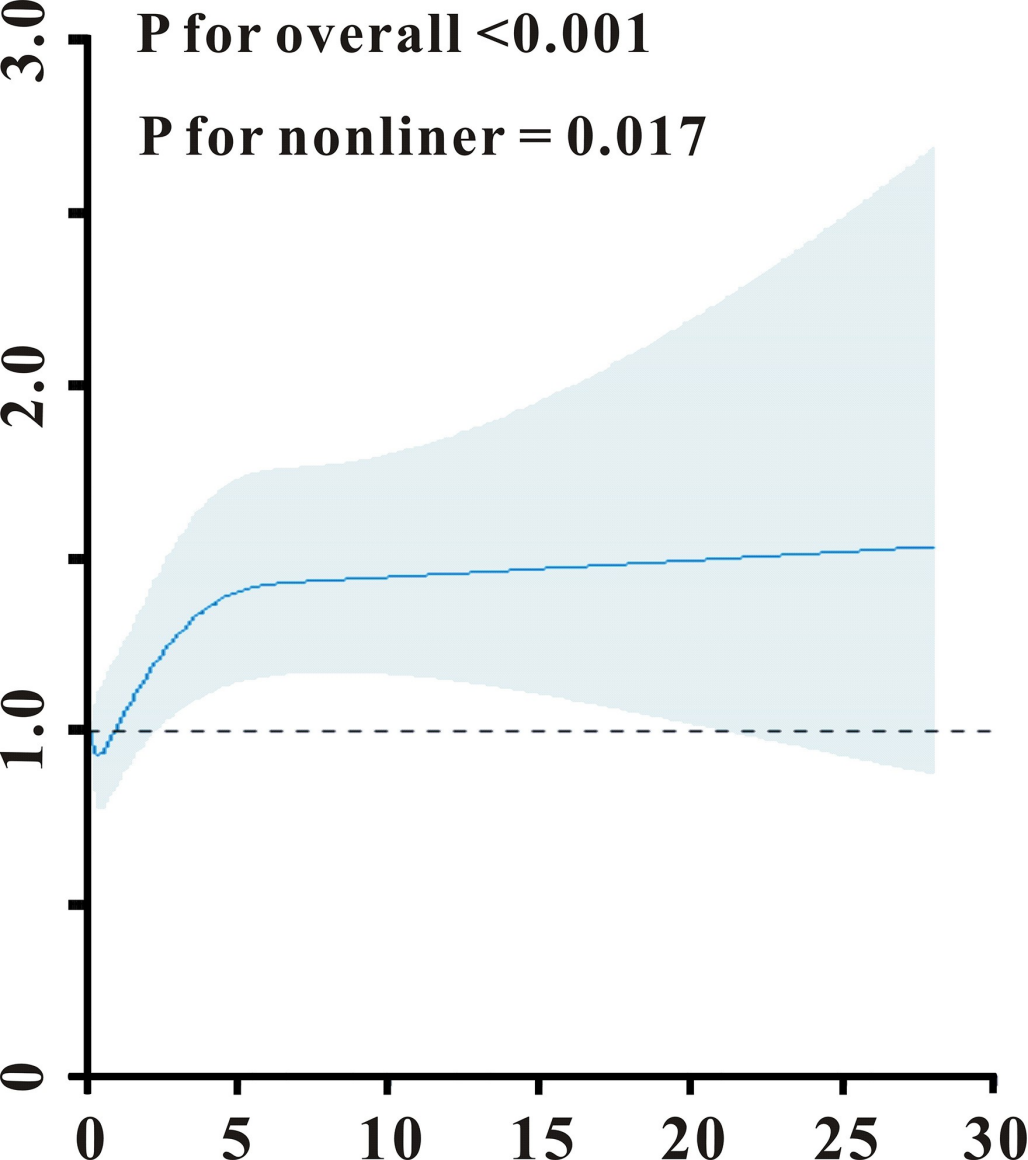
Association between SIRI and AKI using a restricted cubic spline regression model in the training set.

**Fig 3:**
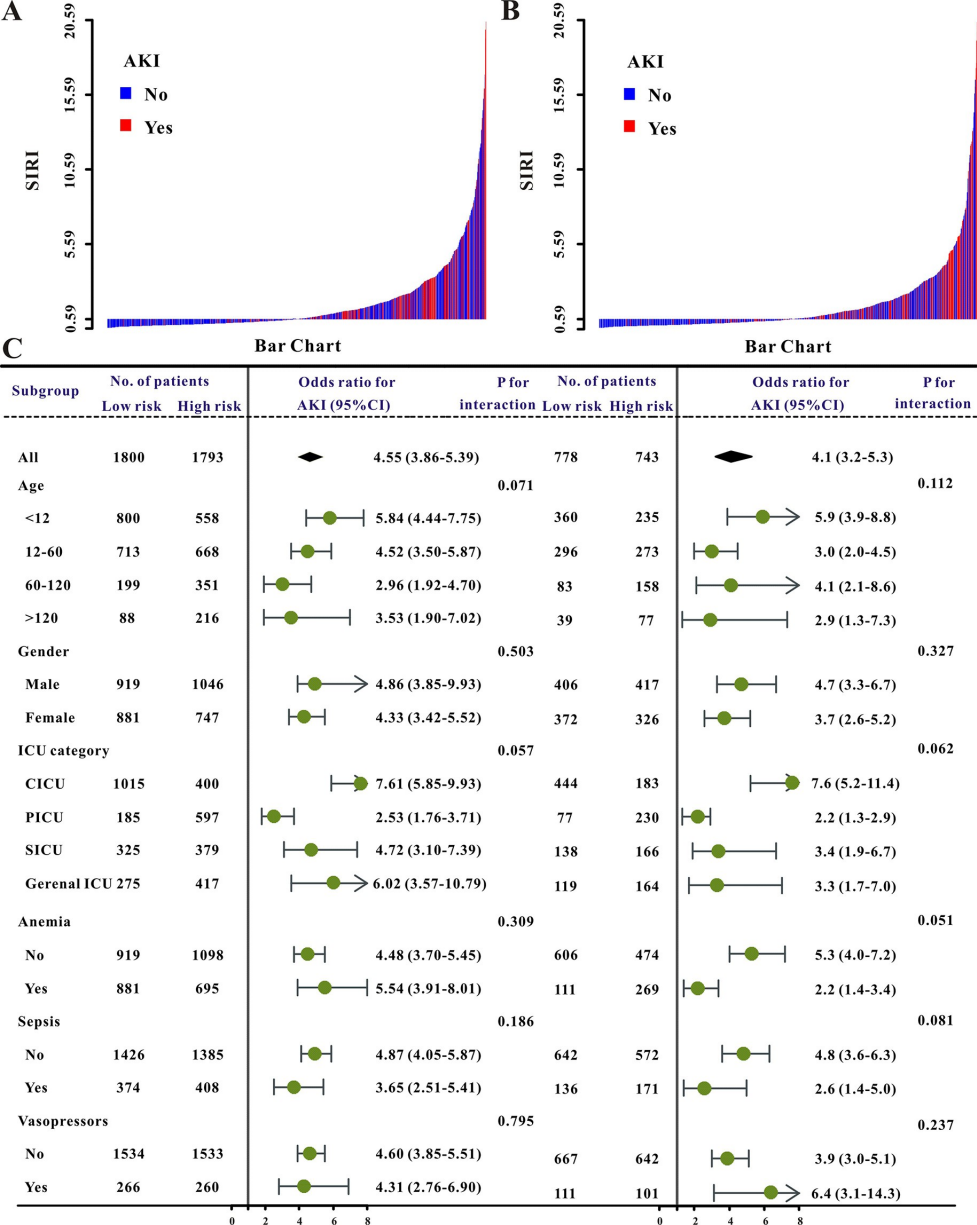
The waterfall plots and forest plots of the high-SIRI group and low-SIRI group for AKI in pediatric patients. The waterfall plot of SIRI for each patient of AKI in the training set (**A**), in the validation set (**B**), and the subgroup analysis of the SIRI for AKI in pediatric patients in both sets (**C**).

The initial SIRI level and the risk of AKI were shown to have a nonlinear connection (P = 0.017 for nonlinearity, Fig 2). After controlling for all possible variables in this investigation, the frequency of AKI raised with SIRI amount when it was larger than 0.59. The incidence of AKI achieved a plateau when the SIRI score was less than 0.59. As a result, each individual was further classified as having a low or high SIRI (< 0.59 or ≥ 0.59). In both the training and validation sets, participants in the reduced SIRI class had a decreased risk of AKI than children in the euphoria SIRI group (Fig 3A and 3B). Furthermore, despite controlling for other clinical factors (age, gender, ICU category, comorbidities as well as laboratory results), SIRI stuck powerful for incidence AKI (odds ratio, OR = 3.95, 95% CI 2.91–5.36, P0.001) in the training set (Table 2). Furthermore, in practically all pre-specified subgroup comparisons, individuals with SIRI < 0.59 had less chance of AKI compared with those with SIRI ≥ 0.59 group in almost all subgroups (Fig 3C). Equivalent results were discovered in the verification cohort (Table 2 and Fig 3C).

**Table 2:** Logistic regression analysis of high SIRI and low SIRI group for AKI.

Methods	HR (95%CI)	P value
In training set		
Unadjusted	4.17 (3.08–5.65)	<0.001
Adjusted for model I	4.05 (2.99–5.48)	<0.001
Adjusted for model II	4.09 (3.02–5.54)	<0.001
Adjusted for model III	3.95 (2.91–5.36)	<0.001
In validation set		
Unadjusted	7.27 (3.62–16.72)	<0.001
Adjusted for model I	7.25 (3.15–16.66)	<0.001
Adjusted for model II	6.62 (2.86–15.29)	<0.001
Adjusted for model III	7.23 (3.78–13.83)	<0.001

AKI, acute kidney injury, HR, hazard ratio, 95%CI, 95% confidence index, Model I adjusted for age, gender, ICU category. Model II adjusted for model I plus comorbidities. Model III adjusted for model II plus laboratory results.

### SIRI as a predictor for secondary outcomes

**Fig 4:**
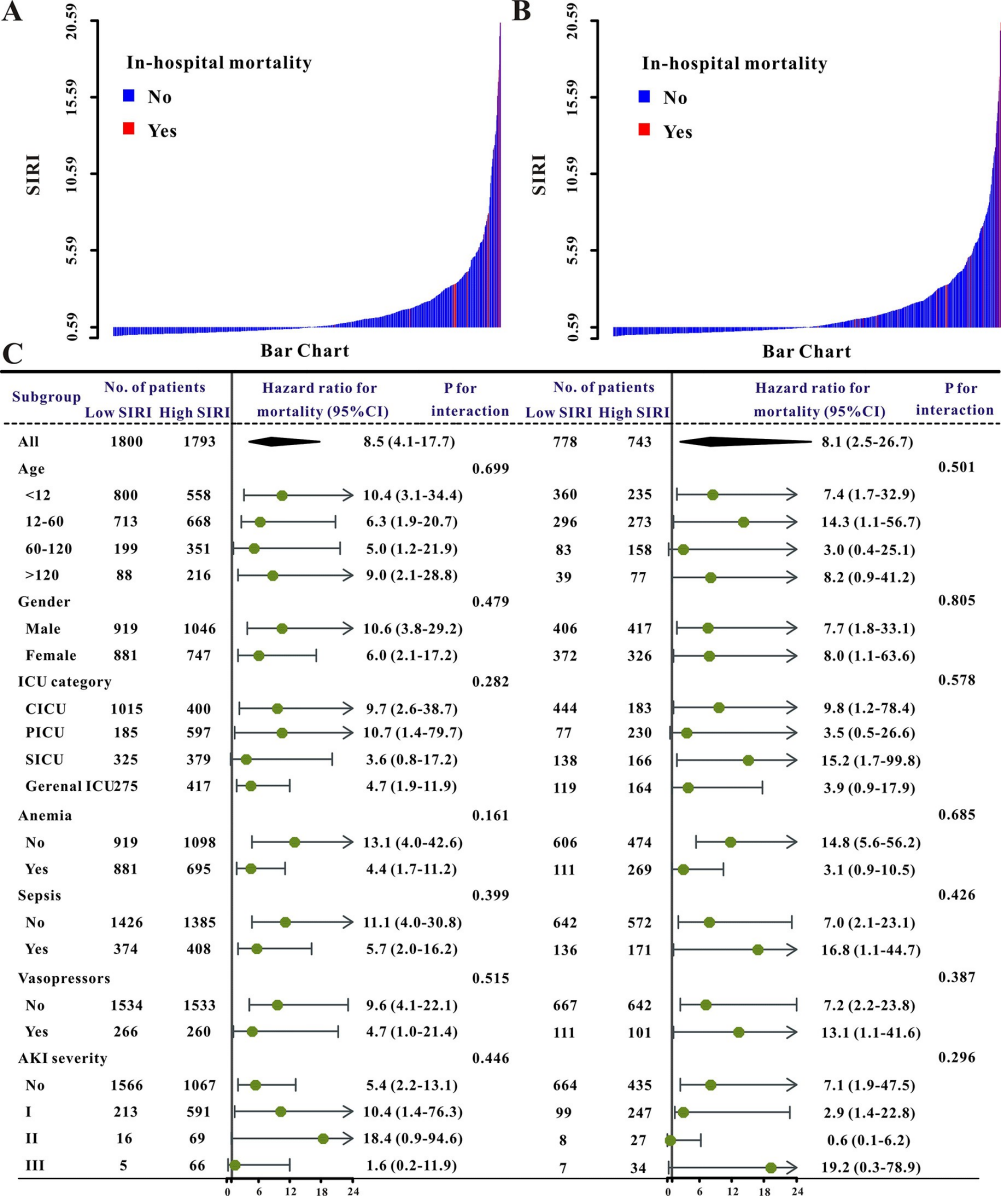
The waterfall plots and forest plots of the high-SIRI group and low-SIRI group for in-hospital mortality in pediatric patients. The waterfall plot of SIRI for each patient of in-hospital mortality in the training set (**A**), in the validation set (**B**), and the subgroup analysis of the SIRI for in-hospital mortality in pediatric patients in both sets (**C**).

**Fig 5:**
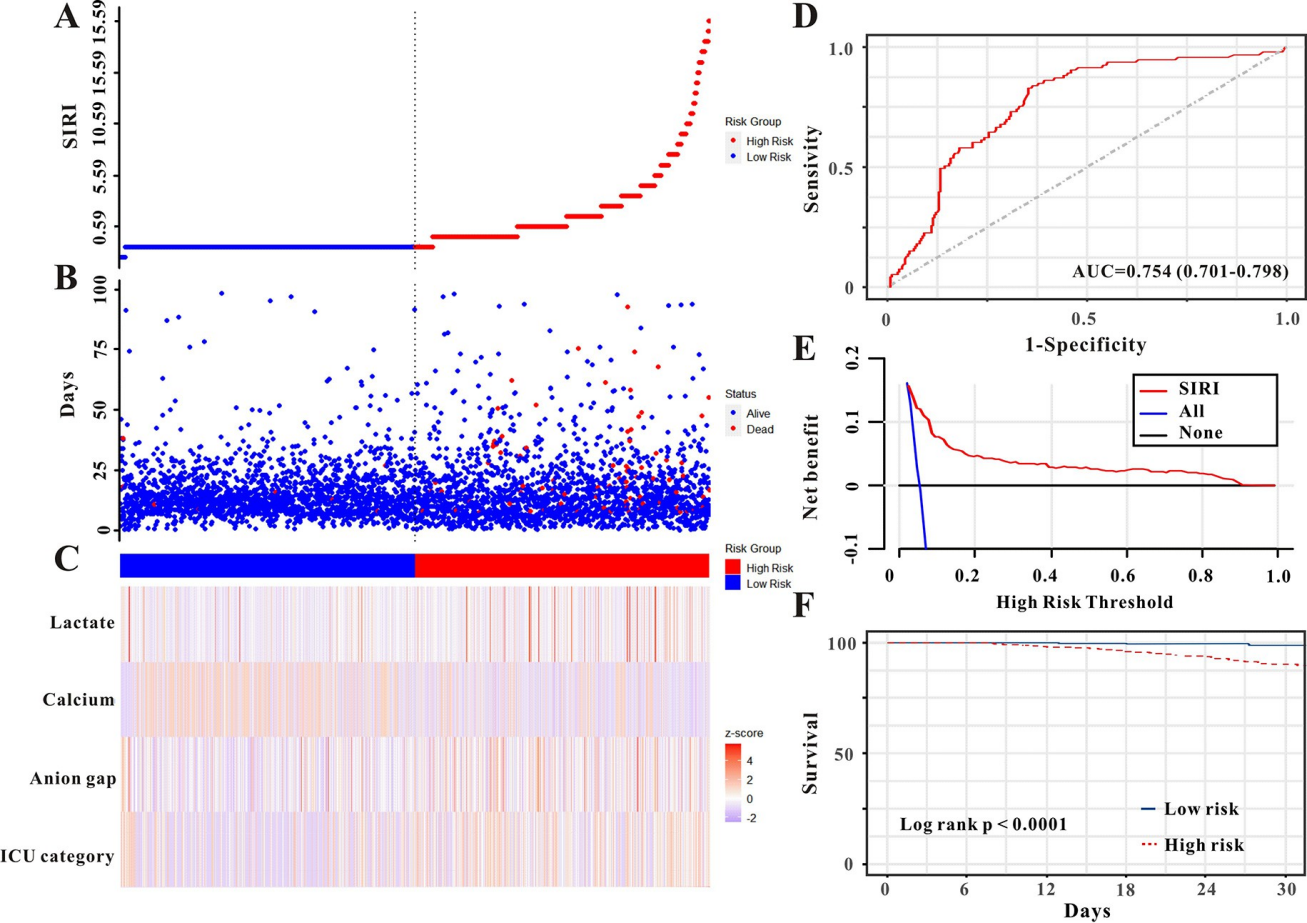
The SIRI was established to detect the in-hospital mortality of patients in pediatric intensive care units in the training set. All patients were distinguished into high and low risk based on the SIRI (**A**), the relationship between survival time and prognosis of patients in the two corresponding groups (**B**), and the heatmap of other markers between the two groups (**C**). Receiver operating characteristic (ROC) curve analysis of the SIRI for overall mortality (**D**), Decision curve analysis of the risk score for the overall mortality (**E**). Kaplan-Meier curves show the overall mortality of groups with different risks (**F**).

Patients in the high SIRI group had a larger frequency of AKI stage II or III, sepsis, and in-hospital mortality, as well as a longer period of ICU and hospital stay (Table 3). Fig 1 illustrates the association between SIRI and clinical variables as well as clinical outcomes in both sets. The SIRI value for each patient in both groups, given as a waterfall plot, revealed substantial differences between patients who survived and died in the hospital (P<0.001, Fig 4A and 4B). Based on the SIRI value of 0.59, patients were classified as high-risk or low-risk for in-hospital mortality (Fig 5A–5C). SIRI’s AUC for in-hospital mortality was 0.754 (Fig 5D). SIRI was demonstrated to be clinically beneficial for doctors using decision curve analysis (DCA) (Fig 5E). For patients in PCIU, the high SIRI group had a poorer outcome than the lower SIRI group (Fig 5F, P<0.0001). Similar findings were discovered in the validation cohort (Table 3 and S2 Fig).

**Table 3:** Clinical outcomes analysis of high and low SIRI groups for all patients.

Outcomes	Low SIRI group	High SIRI group	Effect size	P value
In training set				
N	1800	1793	-	-
AKI	234 (13.0)	726 (40.5)	0.653	<0.001
AKI severity			0.665	<0.001
Stage I	213 (91.0)	591 (81.4)		
vStage II	16 (6.8)	69 (9.5)		
Stage III	5 (2.2)	66 (9.1)		
Usage of vasopressors	266 (14.8)	260 (14.5)	0.034	0.590
Sepsis	344 (19.1)	438 (24.4)	0.076	0.025
Length of ICU stay	1.9 (0.9, 8.9)	4.6 (1.6, 10.5)	0.378	<0.001
Length of hospital stay	13.1 (8.9, 19.0)	13.1 (7.8, 20.9)	0.083	0.013
In-hospital mortality	8 (0.4)	85 (4.7)	0.273	<0.001
In validation set				
N	778	743	-	-
AKI	114 (14.7)	308 (41.5)	0.625	<0.001
AKI severity			0.630	<0.001
Stage I	99 (12.7)	247 (33.2)		
Stage II	8 (1.0)	27 (3.6)		
Stage III	7 (0.9)	34 (4.6)		
Usage of vasopressors	111 (14.3)	101 (13.6)	0.019	0.760
Sepsis	136 (17.5)	171 (23.0)	0.138	0.009
Length of ICU stay	1.9 (0.9, 4.0)	3.8 (1.1, 9.8)	0.367	<0.001
Length of hospital stay	13.1 (9.0, 19.0)	13.7 (8.0, 21.1)	0.169	0.001
In-hospital mortality	3 (0.4)	33 (4.4)	0.267	<0.001

AKI, acute kidney injury, ICU, intensive care unit, SIRI, systemic inflammatory response index, BUN, blood urea nitrogen.

The high SIRI patients had a higher death rate, with a crude hazard ratio (HR) of 4.17 (95% CI, 3.08–5.65, P0.001), and the link remained robust after controlling for other risk factors (age, gender, ICU category, comorbidities as well as laboratory results, Table 4). In practically all subgroups of the pre-specified subgroup analysis, individuals with SIRI < 0.59 had a reduced probability of in-hospitalization than those with SIRI ≥ 0.59 group in almost all subgroups (Fig 4C). Furthermore, patients in the validation groups had similar results (Table 4 and Fig 4C), indicating that SIRI had an independent effect on PICU patients.

**Table 4:** COX regression analysis of high SIRI and low SIRI group for in-hospital mortality.

Methods	HR (95%CI)	P value
In training set		
Unadjusted	8.54 (4.13–17.68)	<0.001
Adjusted for model I	7.31 (3.53–15.14)	<0.001
Adjusted for model II	5.09 (2.41–10.73)	<0.001
Adjusted for model III	5.01 (2.09–12.03)	<0.001
In validation set		
Unadjusted	8.13 (2.47–26.70)	0.001
Adjusted for model I	7.30 (2.22–24.06)	0.001
Adjusted for model II	4.26 (1.25–14.49)	0.020
Adjusted for model III	3.58 (1.02–12.60)	0.047

AKI, acute kidney injury, HR, hazard ratio, 95%CI, 95% confidence index, Model I adjusted for age, gender, ICU category. Model II adjusted for model I plus comorbidities, usage of vasopressors, and AKI. Model III adjusted for model II plus laboratory results.

### Development and verification of the predictive nomogram

**Fig 6:**
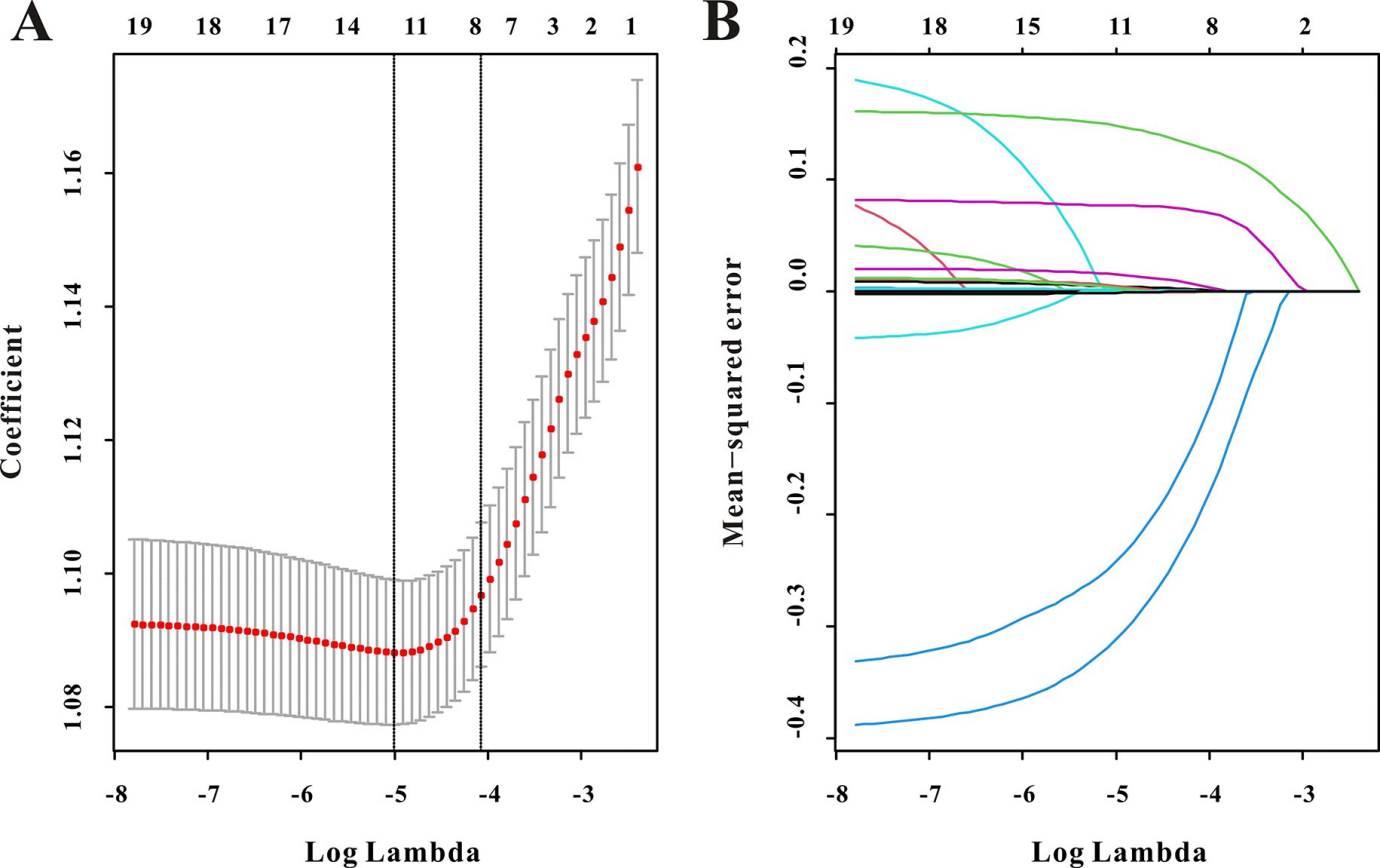
Selection of significant factors associated with AKI in pediatric patients by LASSO regression model.

**Fig 7:**
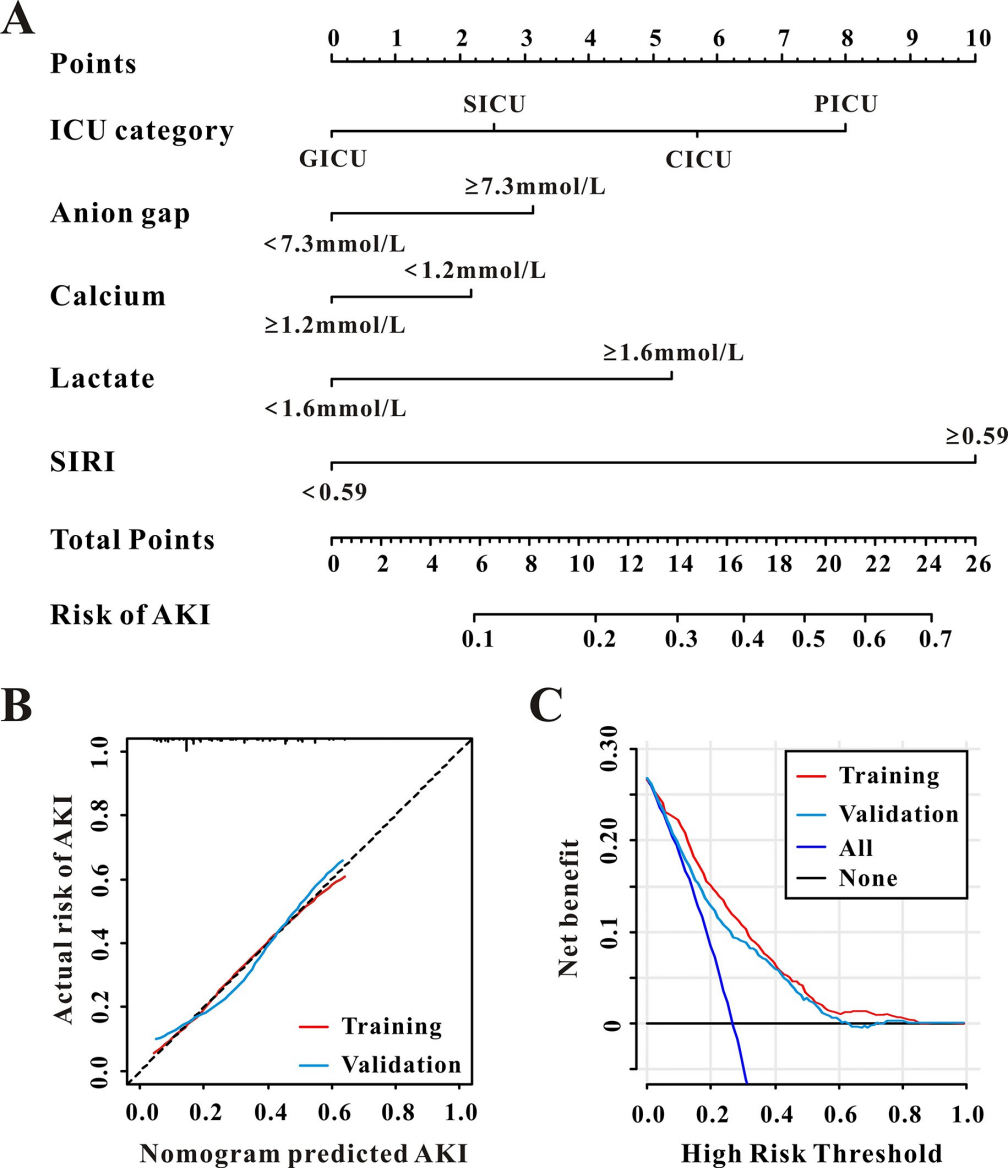
The established nomogram for AKI in pediatric patients (**A**), The calibration curves (**B**), and the decision curve analysis (**C**) of the nomogram for AKI in the training set and the validation set.

This study comprised eight components, according to LASSO regression for AKI (Fig 6A and 6B). Furthermore, multivariate logistic analysis was utilized to construct a nomogram for AKI in PICU patients. As demonstrated in Table 5, this nomogram now includes the ICU category, anion gap, calcium, lactate, and SIRI (Fig 7A).

**Table 5:** Logistic regression analysis for the factors of AKI selected by LASSO regression in the training set.

	Univariate		Multivatiate	
	OR (95% CI)	P	OR (95% CI)	P
ICU category				
CICU	1.52 (1.21–1.90)	<0.001	2.85 (2.14–3.81)	<0.001
PICU	2.91 (2.29–3.70)	<0.001	2.88 (2.24–3.71)	<0.001
SICU	1.21 (0.93–1.58)	0.151	1.53 (1.14–2.03)	0.004
GICU	Ref.	-		
Hemoglobin	1.01 (1.00–1.02)	0.054		
Platelets	0.99 (0.97–0.99)	<0.001	1.00 (0.98–1.09)	0.068
SIRI	1.17 (1.14–1.21)	<0.001	1.17 (1.13–1.20)	<0.001
Total bilirubin	1.01 (1.00–1.01)	0.012	1.00 (0.91–1.24)	0.124
Anion gap	1.03 (1.02–1.05)	<0.001	1.02 (1.01–1.14)	0.010
Calcium	0.71 (0.63–0.80)	<0.001	0.82 (0.69–0.97)	0.018
Lactate	1.13 (1.09–1.17)	<0.001	1.08 (1.04–1.13)	<0.001

OR, odds ratio, 95%CI, 95% confidence index, CICU, cardiac intensive care unit, PICU, pediatric intensive care unit; SICU, surgery intensive care unit, GICU, general intensive care unit, SIRI, systemic inflammatory response index, BUN, blood urea nitrogen

In terms of performance, the Brier score for this predictive nomogram in the training and validation sets was 0.128 and 0.132, respectively, indicating rather good performance. In all sets, the calibration curves showed excellent agreement between anticipated and actual AKI (Fig 7B). Furthermore, DCA indicated that the prediction nomogram was useful in both sets for decision-making (Fig 7C).

## Discussion

In this study, we discovered a strong relationship between initial SIRI, AKI, and mortality for children who were treated in the PICU. In practically all categories, 0.59 was a suitable threshold for the probability of AKI and patient prognosis. We then created a prediction nomogram for AKI that incorporates SIRI and other clinical variables and has excellent calibration, prejudice, and clinical relevance. Taking these findings together, we believe that preliminary SIRI was a credible marker for AKI and the prognosis of pediatric patients in the PICU, and that the suggested nomogram utilizing SIRI produced a reliable estimate for the identification of pediatric patients with AKI.

Despite the reality that AKI is one of the most prevalent complications in adult or pediatric ICU patients that results in poorer clinical outcomes, longer hospital stays, and higher healthcare costs, AKI in pediatric patients has still been undervalued, due in part to different diagnostic criteria, an absence of indicators for early identification, and a lack of markers for early referral [17, 18]. Unlike the KDIGO-AKI criteria, the pROCK-AKI criteria were developed in China based on pediatric hospitalized patients and the idea that only an abrupt rise in SCr over the upper limit of normal variability signifies a real deterioration in renal function [16]. According to the pROCK criteria, the overall frequency of AKI for pediatric patients in the ICU was 27.0% in this study, which was higher than in prior studies and may be explained in part by the severity of the disease and the varied AKI criteria. Aside from the significant morbidity of AKI, the scarcity of viable biomarkers for early detection is another reason why so many researchers have focused on it. As a result, the objective for those patients is to utilize model predictions to act early and avoid the onset of AKI.

There were several indicators for AKI in various populations that had been investigated in prior research throughout the decades; however, significant drawbacks, such as cost and complexity, prevented their clinical use until recently [19, 20]. As a result, an accurate, simple, and cost-effective AKI biomarker is critical for doctors to identify at-risk patients and focus the deployment of renal protective measures. Among all the indicators, WBC-based ratios, such as neutrophil to lymphocyte ratio, had high hopes due to their benefits of being easy to acquire, cost-effective, and somewhat reliable, and some of them were even used in clinics in select hospitals [21, 22]. SIRI is another one of the most effective WBC-based indicators, and prior studies have demonstrated a link between early SIRI and clinical outcomes in patients with various illnesses. SIRI has also been shown to have prognostic significance for renal cell carcinoma patients [10]. Furthermore, a recent investigation found that SIRI was a unique predictor of overall survival in diabetes patients on maintained hemodialysis [23]. SIRI was a valid index not only for patients with chronic renal disease but also for those with acute kidney illness. Yang et al. carried out a longitudinal investigation of 950 clients employing data from acute coronary syndrome patients undergoing coronary angioplasty and decided that beforehand hematological cytokines, including SIRI, had clinical significance for assessing the likelihood of contrast-induced AKI [12]. Another historical cohort research found that SIRI is a unique and effective predictor of AKI onset and outcome in patients with abdominal trauma [13]. However, this is a novel investigation examining the relationship between inaugural SIRI and clinical consequences in pediatric patients, and we indicated that SIRI might be a novel predictor of AKI and prognosis in children treated in the intensive care unit.

It is widely accepted that there is a distinct relationship between AKI and inflammatory conditions, and because AKI is a form of inflammatory illness of the kidneys, substantial numbers of cytokines and inflammatory agents can be generated during an AKI episode [24]. AKI also provided a slew of inflammatory factors, including tumor necrosis factor (TNF)-α and nucleotide-binding domain-like receptor protein 3 (NLRP3) [25]. Furthermore, as the severity of AKI progresses, the levels of inflammatory factors rise. Previous research has linked elevated TNF-α and NLRP3 levels to the occurrence and development of renal damage, eventually leading to AKI [26, 27]. Furthermore, white blood cells and polymorphisms are the most popular and simple techniques for doctors to identify a patient’s inflammatory status. A high SIRI indicates a high neutrophil and monocyte concentration but a low lymphocyte content. Previous research has shown that higher neutrophil and monocyte numbers, or decreased lymphocyte counts, are effective indicators of renal disease prognosis. Research of 646 peritoneal dialysis patients with a median follow-up of 31 months discovered that a greater degree of SIRI was independently associated with all-cause mortality and CVD mortality [28]. Nonetheless, the specific mechanism by which the SIRI causes AKI in pediatric ICU patients is unknown. We feel that, except for inflammation, they might be explained in part by hunger. This study discovered a substantial negative association between SIRI and serum albumin (r = -0.206, P0.001), and it is well known that starvation is an additional reliable predictor of AKI in both children and adults [29].

This retrospective examination did, however, have several drawbacks. To begin, this retrospective analysis was single-center research with relatively small sample sizes, which might lead to bias. Second, we only recorded the initial SIRI measures and did not assess changes in them throughout their ICU stay. Third, certain critical inflammatory indicators, such as interleukin and tumor necrosis factor, were overlooked in the database. Furthermore, we did not investigate the relationship between SIRI and AKI recovery, development, and other AKI outcomes. Finally, the predicted nomogram was not validated in a separate cohort, and more prospective multicenter investigations are required to confirm our findings.

## Conclusions

In this work, we first determined that the first SIRI, an easy-to-obtain and low-cost measure, might be a reliable predictor of AKI and outcome in critically sick pediatric patients. Furthermore, the proposed nomogram based on the first SIRI is a useful method for identifying individuals at high risk of AKI and may provide a pharmacological stimulus for pediatric patients who are suffering from critical care sickness.

## Supporting information

S1 Fig The correlations between SIRI and other clinical variables and outcomes. (DOCX)

S2 Fig The SIRI was established to detect the in-hospital mortality of patients in pediatric intensive care units in the validation set. (DOCX)
