# Supplementary material for: Prognostic value of systemic inflammatory response index for acute kidney injury and the prognosis of pediatric patients in critical care units
Source: PLoS One. 2024 Aug 29;19(8):e0306884. doi: 10.1371/journal.pone.0306884 (PMC11361669; doi:10.1371/journal.pone.0306884)
Supplement: S1 Fig — (DOCX) [file pone.0306884.s001.docx]

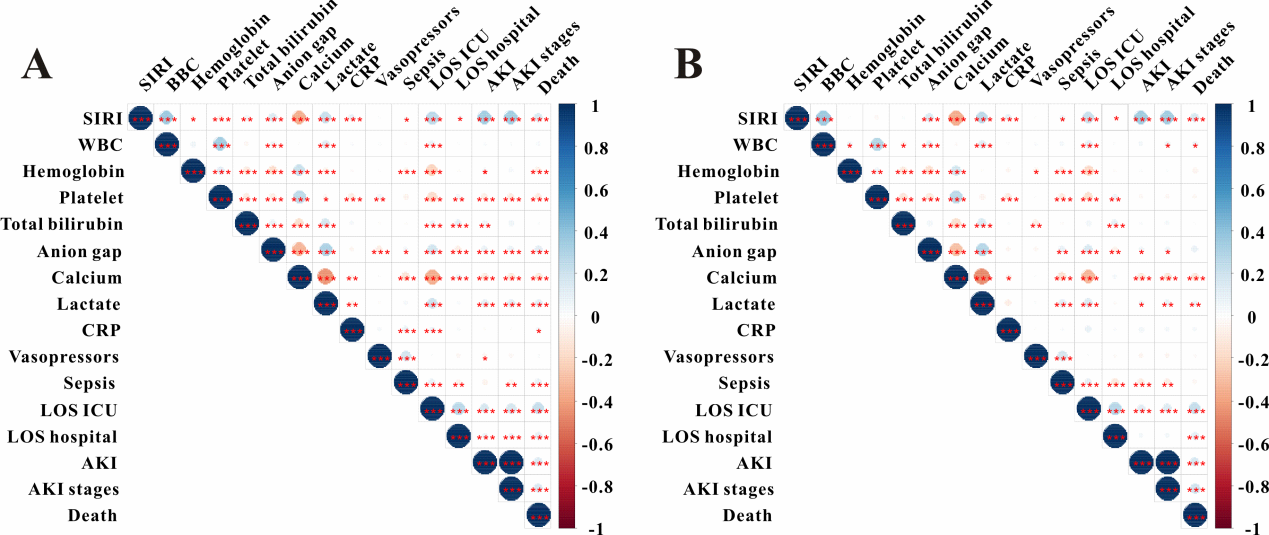


**Supplemental Figure 1** The correlations between SIRI and other clinical variables, and clinical outcomes in the training set (**A**) and the validation set (**B**).
