# Supplementary material for: Prognostic value of systemic inflammatory response index for acute kidney injury and the prognosis of pediatric patients in critical care units
Source: PLoS One. 2024 Aug 29;19(8):e0306884. doi: 10.1371/journal.pone.0306884 (PMC11361669; doi:10.1371/journal.pone.0306884)
Supplement: S2 Fig — (DOCX) [file pone.0306884.s002.docx]

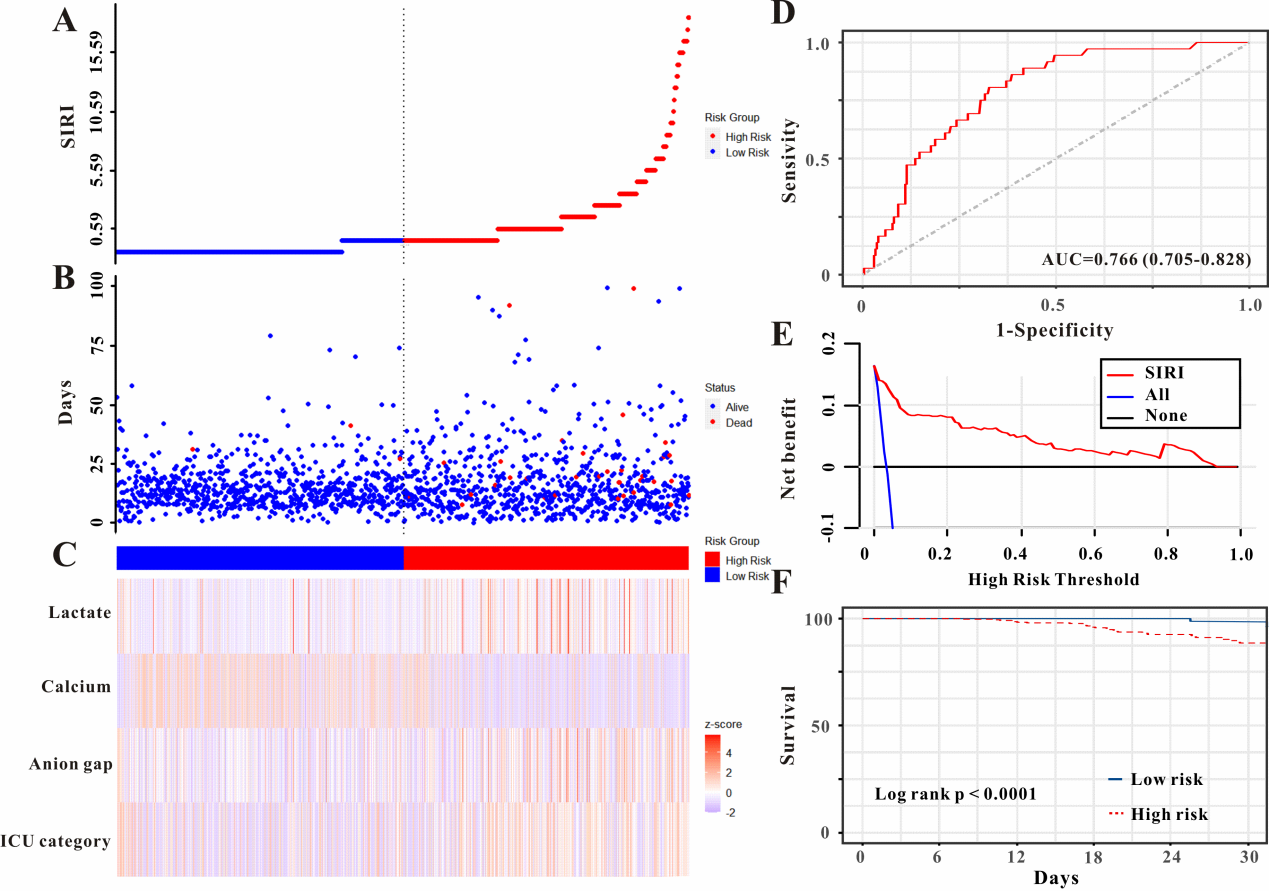


**Supplemental Figure 2** The SIRI was established to detect the in-hospital mortality of patients in pediatric intensive care units in the validation set. All patients were distinguished into high and low risk based on the SIRI (**A**), the relationship between survival time and prognosis of patients in the two corresponding groups (**B**), and the heatmap of other markers between the two groups (**C**). Receiver operating characteristic (ROC) curve analysis of the SIRI for overall mortality (**D**), Decision curve analysis of the risk score for the overall mortality (**E**). Kaplan-Meier curves show the overall mortality of groups with different risks (**F**).
